# Supplementary material for: Investigating the Impact of Food Rewards on Children’s Motivation to Participate in Sport
Source: Children (Basel). 2023 Feb 23;10(3):432. doi: 10.3390/children10030432 (PMC10047004; doi:10.3390/children10030432)
Supplement: Supplementary file 1 [file children-10-00432-s001.zip › Supplementary file S1_Themes and Codes.pdf]

Inductive Themes and Codes

Themes	Subthemes	Codes
**The Culture of Food in Soccer and Hockey**	Venue Food<br>Team Snacks<br>Time Constraints and the Convenience of Fast Food	• Types of Food<br>• Expectation of certain types<br>• Utilized<br>• Food in Soccer<br>• Food in Hockey<br>• Post-Soccer Food<br>• Post-Hockey Food<br>• Pre-Soccer Food<br>• Pre-Hockey Food<br>• Tournament Food
**The How and Why of Motivating Child Athletes**	Rewards<br>Rationale for Rewards<br>(Un)importance of Rewarding<br>Tradition<br>Intrinsically Motivated Children<br>Athlete's Favorite Part of Soccer and Hockey	• Rewards in Soccer<br>• Rewards in Hockey<br>• Motivating Children/Athletes<br>• Game vs Practice Motivation<br>• Parents Motivating Athletes<br>• Importance of Treats<br>• Importance of Rewards<br>• Participation without Rewards<br>• Rationale for Rewards<br>• Child's Participation Rationale
**Opinions of Sponsorship in Youth Sport**	Financial Support<br>Appropriateness	• Opinion<br>• Appropriateness<br>• Community Involvement<br>• Monetary Support<br>• No opinion
