# Supplementary figures and images for: Investigating the Impact of Food Rewards on Children’s Motivation to Participate in Sport
Source: Children (Basel). 2023 Feb 23;10(3):432. doi: 10.3390/children10030432 (PMC10047004; doi:10.3390/children10030432)

## Graphic Elicitation Examples

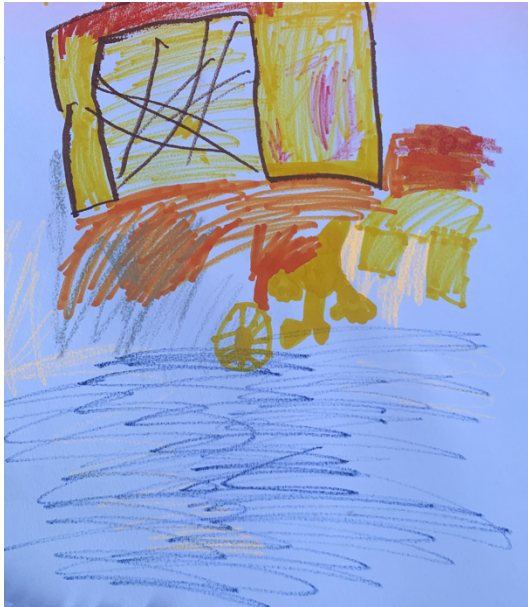

SFG1 A3

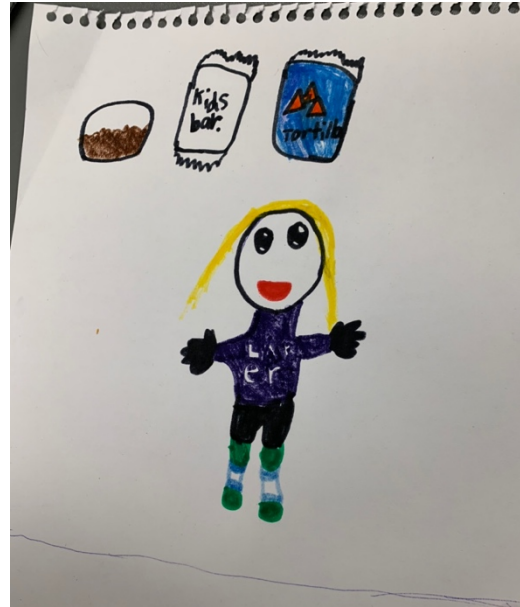

HFG6 A4

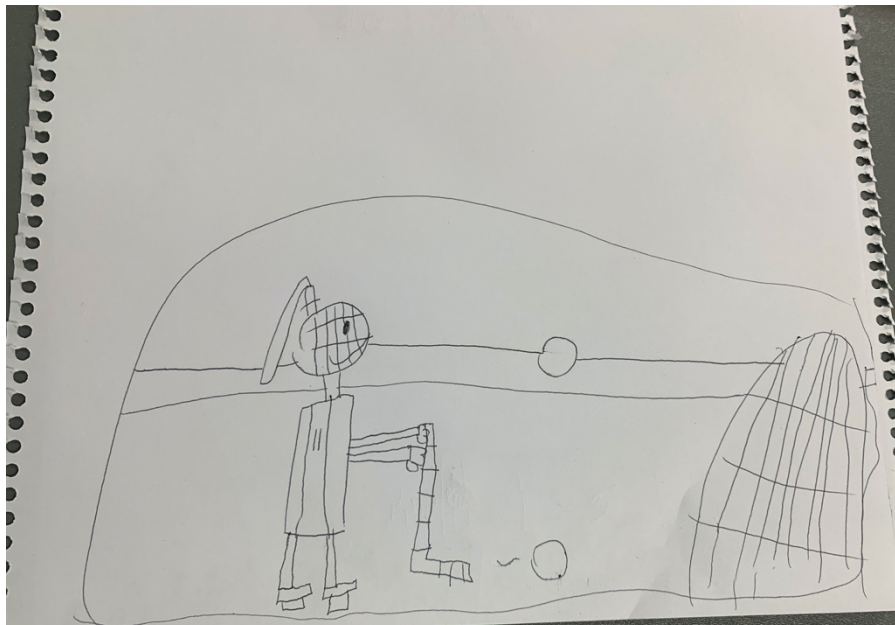

HFG6 A1

Supplement: Supplementary file 1 [file children-10-00432-s001.zip › Supplementary file S2_Graphic Elicitation Examples.pdf]
